# THE FINANCIAL AND EMOTIONAL IMPACT OF COVID-19 FOR DEMENTIA CAREGIVERS: A COMPARISON OF THE NHATS AND HISPANIC EPESE

**DOI:** 10.1093/geroni/igad104.3642

**Published:** 2023-12-21

**Authors:** Sunshine Rote, Jacqueline Angel, Phillip Cantu, Felipe Antequera

**Affiliations:** University of Louisville, Louisville, Kentucky, United States; University of Texas at Austin LBJ School, Austin, Texas, United States; The University of Texas Medical Branch, Galveston, Texas, United States; University of Texas at Austin LBJ School, Austin, Texas, United States

## Abstract

During the COVID-19 pandemic, older adults and their caregivers/care partners (hereafter referred to as “caregivers”) experienced social isolation, reduced autonomy, disruptions in medical care and barriers to community-based services such as adult day centers. We compare the extent to which these disruptions affected white, Black, and Hispanic caregivers. We utilize newly released data on adult children caregivers from the Caregiver Supplement to the Hispanic Establish Population for the Epidemiologic Study of the Elderly (HEPESE CG, 2021) and the National Health and Aging Trends COVID-19 Family Members and Friends Dataset (NHATS FF, 2020/2021) to compare caregiver emotional well-being and financial strain among non-Hispanic White (NH White, n=327), non-Hispanic Black (NH Black, n=57), and Mexican American (n=57) adult children caregivers. Results reveal that a larger fraction of NH Black and Mexican American caregivers reported financial strain than NH White caregivers, and that more Mexican American caregivers reported loneliness and depression than other groups of caregivers. For Mexican American caregivers, those reporting lower financial well-being tended to be younger. For NH Black caregivers, there were no significant predictors of financial strain or wellbeing. Finally, NH White caregivers were younger, and more likely to be female and NH White dementia caregivers reported more financial strain and loneliness than NH White non-dementia caregivers. Taken together, this work provides valuable insight into the role of the COVID-19 pandemic in exacerbating caregiver health disparities among caregivers and provides valuable insight as well as to the high-impact intervention points for caregivers from minoritized populations.

